# Clinical study on the safety and efficacy of remimazolam in hysteroscopic surgery under general anesthesia in elderly patients

**DOI:** 10.3389/fmed.2024.1409233

**Published:** 2024-11-07

**Authors:** Manjie Xie, Fanrui Zeng, Qiao Tian, Huiwei Deng, Shanqing Tao

**Affiliations:** ^1^Department of Anesthesiology, Changde Hospital, Xiangya School of Medicine, Central South University (The First People's Hospital of Changde City), Changde, China; ^2^Department of Orthopedic Surgery, Changde Second People's Hospital, Changde, China; ^3^Department of Infectious Diseases, Changde Hospital, Xiangya School of Medicine, Central South University (The First People's Hospital of Changde City), Changde, China

**Keywords:** remimazolam, old age, hysteroscopic surgery, clinical study, general anesthesia

## Abstract

**Objective:**

To evaluate the safety and efficacy of remimazolam in hysteroscopic surgery in elderly patients.

**Methods:**

Following hysteroscopic surgery under selected general anesthesia, 60 elderly patients ASA (American Society of Anesthesiologists) class II–III, >65 years old were randomly assigned to one of two groups: the R group (remimazolam) or the C group (propofol), each with 30 patients. Sufentanil 0.1 μg/kg was given 5 min before the operation, remimazolam 0.2 mg/kg intravenously in Group R, then 0.5~1 mg/(kg.h) by pump, propofol 2 mg/kg intravenously in group B, and then 4~8 mg/(kg.h) by pump. Maintain BIS (Bispectral index) 40~70, add remimazolam 0.05 mg/kg or propofol 0.5 mg/kg when the patient is in motion, and stop the administration at the end of the operation. Record the patients' HR, MAP, RR, SpO2, PETCO2, and BIS values at entry (T0), before induction administration (T1), 1 min after administration (T2), 5 min after administration (T3), when stopping administration (T4), when awakening (T5), and 1 min after awakening (T6), as well as the onset time after administration, the awakening time, the success rate of sedation, and the number and dose of additional medications. Reactions are adverse (hypotension, hypertension, respiratory depression incidence, injection pain, nausea and vomiting following surgery, etc.).

**Results:**

The two groups' respective anesthetic success rates were comparable overall. In addition to having a higher BIS value and more extra medications than group C, group R experienced less incidence of respiratory depression, injection pain, and intraoperative hypotension.

**Conclusion:**

Remimazolam, which is equivalent to propofol in terms of safety and efficacy for older patients undergoing hysteroscopic surgery, should be further promoted and used.

## 1 Introduction

As a minimally invasive procedure offering diagnostic and therapeutic benefits, hysteroscopy has emerged as the foremost method for diagnosing and treating gynecological conditions (1). With the aging demographic, a growing number of elderly individuals are undergoing hysteroscopic surgeries. However, due to age-related declines in organ function and the presence of comorbidities, administering anesthesia to elderly patients presents significant challenges. Currently, propofol intravenous general anesthesia stands as the most prevalent method for hysteroscopic surgeries (2). Nonetheless, its potent respiratory and circulatory depressant effects entail certain risks when utilized in elderly patients (3). The novel benzodiazepine intravenous anesthetic Remimazolam is fast-acting, has a short duration, and help in rapid recovery (4, 5). Metabolized by plasma esterases, it exerts minimal impact on hemodynamics and can be swiftly counteracted by flumazenil. In principle, it represents an ideal anesthetic choice for elderly patients undergoing hysteroscopic surgery (6–9). The aim of the study is to determine whether remimazolam is safe and effective for use during general anesthesia for hysteroscopic surgery in elderly patients. It also advocates for standardized clinical application protocols and provides insights for judicious medication usage in this age group.

## 2 Materials and methods

### 2.1 General information

All patients gave their signature on the informed consent form, which was approved by the hospital's ethics committee. Prior to surgery, patients were regularly instructed to abstain from eating and drinking. Additionally, upon arrival in the operating room, patients were given oxygen. A total of 60 elderly patients aged 65 to 80 years undergoing scheduled hysteroscopic surgery from June 2021 to May 2022 were selected. They were classified as ASA II to III, with an expected surgery duration of 10 to 30 min. Exclusion criteria included patients allergic to remimazolam or any of its components, uncontrolled severe hypertension, unstable angina, severe liver, kidney, or respiratory dysfunction, history of prolonged sedative or analgesic drug use, allergic history, patient refusal, and other conditions deemed unsuitable by the researchers. These patients were divided into two groups at random, with 30 cases in each group: the control group (propofol, or C group) and the experimental group (remimazolam, or R group).

### 2.2 Research methods

Prior to surgery, patients were usually fasted, and they were given oxygen when they were admitted to the operating room. Their electrocardiogram and Bispectral Index (BIS) were monitored. Patients received a 0.1 μg/kg intravenous injection of sufentanil during surgical disinfection. Anesthesia induction protocol: The experimental group was given an intravenous injection of remimazolam at a dose of 0.3 mg/kg 1 min prior to surgery. Thereafter, they were continuously infused with 0.6 mg/(kg·h) until the start of the surgery or until BIS < 65. If BIS >65, remimazolam could be supplemented with a 0.1 mg/kg dose once. If BIS remained >65 after supplementation, propofol 0.5 mg/kg could be added and recorded as a failure. The control group was given an intravenous injection of propofol at a dose of 2 mg/kg, and then received a continuous infusion of 4 mg/(kg·h); 0.5 mg/kg of propofol might be added if the patient's BIS was >65. Anesthesia maintenance: Remimazolam 0.1 mg/kg was given as a supplemental dosage to the experimental group if the patient moved or if their BIS > 65, while propofol 0.5 mg/kg was given to the control group. If BIS remained >65 after two supplementations in the experimental group, propofol 0.5 mg/kg could be added and recorded as a failure. The infusion was stopped at the end of the surgery to facilitate a natural waking. Patients with SpO_2_ < 92% received jaw support, and symptomatic treatment was administered for significant fluctuations in heart rate and blood pressure.

### 2.3 Observation indicators

Safety indicators were monitored at several points in time, such as the time of admission, before induction, 1 min after drug administration, 5 min after drug administration, at the end of drug administration, upon awakening, and 10 min after awakening. Heart rate (HR), respiratory rate (RR), mean arterial pressure (MAP), and pulse oxygen saturation (SpO_2_) were among these indications (T0–6). Additionally, awakening time, occurrences of adverse reactions (post-operative nausea and vomiting, dizziness, injection pain), and instances of jaw support were documented. Observer's Assessment of Alertness/Sedation (OAA/S) scores and the Bispectral Index (BIS) were among the effectiveness indicators that were recorded at the same time points as safety indicators. The anesthesia success rate, number of movements, and instances of drug supplementation were also documented. All data were collected by an experienced anesthesiologist blinded to the group assignments.

### 2.4 Statistical methods

SPSS 26.0 software was utilized to conduct the statistical analysis. Groups were compared using one-way analysis of variance, and normally distributed continuous variables were displayed as mean ± standard deviation. Within-group comparisons were carried out at different times using repeated measures analysis of variance for continuous data. With a significance level of *p* < 0.05, between-group comparisons involving frequency data expressed as counts were performed using either the chi-square test or Fisher's exact probability test.

## 3 Results

Both groups of patients successfully completed the surgery without any serious complications occurring. The two patient groups' general conditions did not significantly differ from one another (Table 1).

**Table 1:** Comparison of general conditions between the two groups of patients (*n* = 30, $\bar{x}$ ± s).

**Group**	**Age (years)**	**Body mass index**	**Surgery duration (min)**
Group R	70.2 ± 6.3	22.6 ± 4.5	16.2 ± 4.3
Group C	71.8 ± 6.7	23.1 ± 6.2	15.3 ± 4.1

*p* > 0.05 for intergroup comparison.

Comparison of HR, MAP, RR, and SpO_2_ at various time points between the two groups of patients: Compared to T_0_, HR decreased at T_2 − 4_ in the experimental group, while in the control group, HR increased at T_2_ and decreased at T_3 − 4_, with both groups recovering to preoperative levels at T_5_. MAP decreased at T_2 − 4_ in both groups, recovering to preoperative levels at T_5_. RR in the control group decreased at T_2 − 3_ and returned to preoperative levels at T_4_. In the experimental group, RR was higher at T_2_, MAP was higher at T_2 − 4_, and HR was lower at T_2_ than in the control group. These differences in intergroup comparison were statistically significant. For each indicator, there were no statistically significant differences in the intergroup comparison at other time points (Table 2).

**Table 2:** Comparison of HR, MAP, RR, and SpO_2_ at various time points between the two groups of patients (*n* = 30, $\bar{x}$ ± s).

**Item**	**Group**	**T_0_**	**T_1_**	**T_2_**	**T_3_**	**T_4_**	**T_5_**	**T_6_**
HR (bpm)	Group R	75 ± 12	71 ± 9	65 ± 6^*#^	67 ± 7^*^	68 ± 8^*^	72 ± 11	73 ± 13
	Group C	76 ± 14	71 ± 10	86 ± 8^*^	64 ± 5^*^	69 ± 8^*^	71 ± 10	72 ± 11
MAP (mmHg)	Group R	112 ± 25	108 ± 23	97 ± 15^*#^	99 ± 18^*#^	105 ± 20^*#^	109 ± 21	112 ± 23
	Group C	110 ± 22	107 ± 21	86 ± 9^*^	84 ± 10^*^	98 ± 16^*^	104 ± 19	111 ± 21
RR (bpm)	Group R	16 ± 3	15 ± 3	13 ± 2^#^	13 ± 3	15 ± 3	16 ± 4	16 ± 3
	Group C	16 ± 3	15 ± 2	10 ± 2^*^	11 ± 3^*^	13 ± 3	15 ± 3	16 ± 2
SpO_2_ (%)	Group R	99 ± 1	100 ± 1	99 ± 1	99 ± 1	100 ± 1	100 ± 1	100 ± 1
	Group C	99 ± 1	100 ± 1	98 ± 1	97 ± 3	98 ± 2	100 ± 1	100 ± 1

^*^Compared to T_0_, *p* < 0.05; ^#^Compared to the control group, *p* < 0.05.

Comparison of the two patient groups' adverse reactions and postoperative awakening times: There were statistically significant differences in the injection pain and jaw support between the R group and the C group. There were no adverse reactions or statistically significant changes in waking times between the two groups (Table 3).

**Table 3:** Comparison of postoperative awakening time and adverse reactions between the two groups of patients (*n* = 30).

**Group**	**Awakening time (min, $\bar{x}$ ±s)**	**Postoperative nausea and vomiting (cases)**	**Dizziness (cases)**	**Injection pain (cases)**	**Jaw support (cases)**
Group R	13.5 ± 4.9	2	4	4^#^	1^#^
Group C	11.6 ± 4.2	1	3	21	15

^#^Compared to the control group, *p* < 0.05.

BIS and OAA/S scores are compared between the two patient groups at different times: Compared to T_0_, both patients showed a decrease in BIS and OAA/S scores during the T_2 − 4_ period, with statistically significant differences. During the T_2 − 3_ period, statistically significant differences were noted in the BIS scores between the R and C groups. At subsequent points, there were not significant differences between the two groups in the other indicators (Table 4).

**Table 4:** Comparison of BIS and OAA/S scores at various time points between the two groups of patients (*n* = 30, $\bar{x}$ ± s).

**Item**	**Group**	**T_0_**	**T_1_**	**T_2_**	**T_3_**	**T_4_**	**T_5_**	**T_6_**
BIS	Group R	95 ± 3	92 ± 3	59 ± 5^*#^	61 ± 5^*#^	62 ± 5^*^	89 ± 7	94 ± 4
	Group C	96 ± 3	93 ± 2	49 ± 4^*^	51 ± 4^*^	55 ± 5^*^	90 ± 6	95 ± 5
OAA/S scores	Group R	5.0 ± 0	5.0 ± 0	0.9 ± 0.3^*^	1.1 ± 0.2^*^	1.6 ± 0.6^*^	4.6 ± 0.5	5.0 ± 0
	Group C	5.0 ± 0	5.0 ± 0	0.8 ± 0.2^*^	0.9 ± 0.2^*^	1.5 ± 0.6^*^	4.8 ± 0.6	5.0 ± 0

^*^Compared to T_0_, *p* < 0.05; ^#^Compared to the control group, *p* < 0.05.

In the experimental group, there was 1 case where remimazolam was supplemented twice intraoperatively, but BIS remained >65. Propofol was added, and BIS dropped to < 60, recorded as one case of anesthesia failure. However, the overall anesthetic success rate did not differ significantly between the two groups. There were noteworthy differences between the R and C groups in terms of the number of intraoperative movements and drug supplementation cases (Table 5).

**Table 5:** Comparison of anesthesia success rate, number of movements, and instances of drug supplementation between the two groups of patients (*n* = 30).

**Group**	**Anesthesia success rate (%)**	**Number of movements**	**Instances of drug supplementation**
Group R	96.7	5^#^	8^#^
Group C	100	1	1

^#^Compared to the control group, *p* < 0.05.

## 4 Discussion

Safety Indicator Analysis: Both groups of patients had similar general conditions. During the T_2_-T_4_ period, both groups experienced varying decreases in HR and MAP. However, the control group exhibited a rapid increase in HR at T_2_, possibly due to increased sympathetic activity after a drop in blood pressure or patient discomfort from injection pain. There were statistically significant intergroup variations in the experimental group's MAP reduction from the control group during the T_2_-T_4_ time points; the experimental group's decrease was much less. Additionally, the control group showed a slight decline in SpO_2_ at T_3_ and T_4_ and a considerable decrease in RR at T_2_. The incidence of injection pain was significantly reduced in the experimental group compared to the control group.

Effectiveness Indicator Analysis: During the T_2_-T_4_ period, both groups achieved effective sedation depths based on BIS and OAA/S scores. More specifically, at T_2_ and T_3_, BIS scores were higher in the experimental group than in the control group, although OAA/S scores were the same. The experimental group had more instances of movement and drug supplementation than the control group, but most were based on increased BIS prompting additional anesthesia, indicating a more sensitive response to BIS. Overall, the anesthesia success rate was comparable between the two groups.

For hysteroscopy operations, propofol is the most commonly utilized intravenous anesthetic because of its rapid onset, short duration of action, and quick recovery. However, it also has significant individual differences and can cause injection pain, respiratory and circulatory depression, propofol infusion syndrome, and dependency on hepatic and renal metabolism, posing risks, especially in elderly patients (10).

The novel ultra-short-acting benzodiazepine sedative-hypnotic agent, remimazolam tosylate, acts on the GABAA receptor, resulting in sedative-hypnotic effects. It has minimal drug interactions, does not accumulate, and is rapidly metabolized by plasma esterases. Remimazolam has a rapid recovery time and onset and offset of action (11), linear pharmacokinetics independent of body weight, and unaffected half-life by infusion time. It can also be rapidly antagonized by flumazenil.

Comparison between Remimazolam Tosylate and Propofol: Remimazolam tosylate is rapidly metabolized without accumulation, while propofol has a longer half-life and can lead to propofol infusion syndrome. Remimazolam tosylate is a safer drug than propofol as it has a lower risk of injection pain, hypotension, and respiratory depression (12, 13). The results are consistent with a previous study (14), but there are the following differences: (1) Different research subjects, we chose elderly (65–80) gynecological surgery patients to provide a basis for their use; (2) Due to different research plans, we focused on the entire surgical process and expanded the scope of use of Remazolam; (3) More comprehensive observation indicators, including objective indicators, scale scores, and all adverse reactions. It has a comparable general anesthesia success rate to propofol in elderly hysteroscopy surgeries, with rapid onset and recovery (15).

## 5 Conclusion

The utilization of remimazolam tosylate in general anesthesia for hysteroscopy surgeries among elderly patients demonstrates commendable safety, exhibiting milder respiratory and circulatory depression compared to propofol and fewer occurrences of adverse reactions such as injection pain. This signifies certain clinical advantages over propofol.

In the context of elderly patients undergoing general anesthesia for hysteroscopy surgeries, remimazolam tosylate showcases rapid onset and recovery while maintaining anesthesia success rates (effectiveness) comparable to propofol. This underscores its potential as an innovative solution for elderly patients undergoing such surgeries and suggests its merit for further promotion and application.

However, limitations of this study include a restricted sample size, which hindered the effective delineation of intergroup differences; insufficient evidence from evidence-based medicine regarding the optimal dosage and regimen of remimazolam tosylate; and a short follow-up period, which impeded a comprehensive assessment of long-term patient outcomes.

## Data Availability

The original contributions presented in the study are included in the article/supplementary material, further inquiries can be directed to the corresponding authors.
